# Case Report: COVID-19-Associated ROHHAD-Like Syndrome

**DOI:** 10.3389/fped.2022.854367

**Published:** 2022-03-31

**Authors:** Irina N. Artamonova, Natalia A. Petrova, Natalia A. Lyubimova, Natalia Yu Kolbina, Alexander V. Bryzzhin, Alexander V. Borodin, Tatyana A. Levko, Ekaterina A. Mamaeva, Tatiana M. Pervunina, Elena S. Vasichkina, Irina L. Nikitina, Anna M. Zlotina, Alexander Yu. Efimtsev, Mikhail M. Kostik

**Affiliations:** ^1^Almazov National Medical Research Centre, Saint Petersburg, Russia; ^2^Saint-Petersburg State Pediatric Medical University, Saint Petersburg, Russia

**Keywords:** ROHHAD-syndrome, obesity, central hypoventilation, hypothalamus dysfunction, hypocorticism, autonomic dysregulation, COVID-19, SARS-CoV-2

## Abstract

It is known that the SARS-CoV-2 virus may cause neurologic damage. Rapid-onset obesity, hypoventilation, hypothalamus dysfunction, and autonomic dysregulation (ROHHAD) syndrome is a disease of unknown etiology with a progressive course and unclear outcomes. The etiology of ROHHAD syndrome includes genetic, epigenetic, paraneoplastic, and immune-mediated theories, but to our knowledge, viral-associated cases of the disease have not been described yet. Here we present the case of a 4-year-old girl who developed a ROHHAD syndrome-like phenotype after a COVID-19 infection and the results of 5 months of therapy. She had COVID-19 pneumonia, followed by electrolyte disturbances (hypernatremia and hyperchloremia), hypocorticism and hypothyroidism, central hypoventilation—requiring prolonged assisted lung ventilation—bulimia, and progressive obesity with hypertriglyceridemia, dyslipidemia, hyperuricemia, and hyperinsulinemia. The repeated MRI of the brain and hypothalamic–pituitary region with contrast enhancement showed mild post-hypoxic changes. Prader–Willi/Angelman syndrome as well as PHOX2B-associated variants was ruled out. Treatment with non-steroidal anti-inflammatory drugs and monthly courses of intravenous immunoglobulin led to a dramatic improvement. Herein the first description of ROHHAD-like syndrome is timely associated with a previous COVID-19 infection with possible primarily viral or immune-mediated hypothalamic involvement.

## Introduction

COVID-19 viral infection may trigger immune dysregulation affecting different targets. It is known that COVID-19 may cause severe neurologic damage, ranging from anosmia, headache, and memory disturbances to several cases of encephalitis (1–3).

Rapid-onset obesity, hypoventilation, hypothalamus dysfunction, and autonomic dysregulation (ROHHAD) syndrome is a rare and complicated condition presenting in previously healthy children. It was first described in 1965 (4, 5). However, its etiology and pathogenesis remain unknown. Usually, it has a progressive course and unclear outcomes. There are several hypotheses about the etiology of this syndrome, including genetic (6–11), epigenetic (7, 12–14), and paraneoplastic theories (4, 15), but autoimmune etiology is becoming more widely discussed lately (6–9, 11–13, 16–24). Viral-associated cases of the ROHHAD syndrome have not been described yet.

Herein we describe the case of a 4-year-old girl who presented with ROHHAD syndrome-like phenotype timely associated with a previous COVID-19 infection. To our knowledge, this is the first description of ROHHAD-like phenotype timely associated with COVID-19 infection.

## Case Description

**Figure 1:**
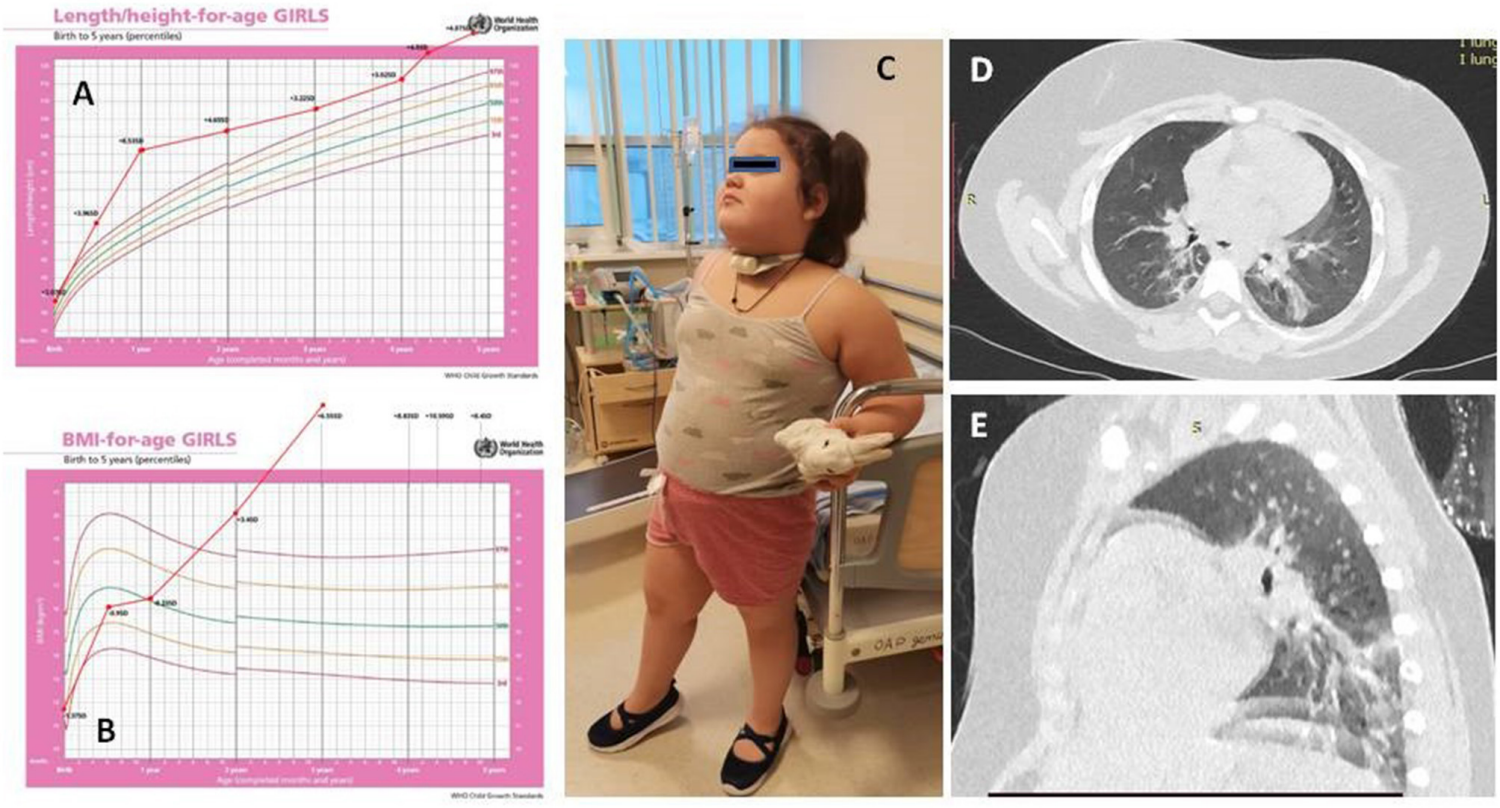
The dynamics of height **(A)** and body mass index **(B)** of the patient. Patient's picture **(C)**. Chest CT **(D,E)**.

A 4-year-old girl was transferred to our hospital with a suspicion of ROHHAD syndrome. She was tall for her age since birth (53 cm, +2.1 SD) and obese since 2 years of age (BMI, 21.0 kg/m^2^, +3.4 SD) but otherwise doing well until 3.5 years (Figures 1A–C).

At 3.5 years, 3 weeks after her family contracted COVID-19, the girl had pneumonia (day 0). Nasal swab PCR for SARS-CoV-2 was not done at this timepoint. She recovered completely after a course of josamycin at 500 mg twice a day for 7 days. After 3 weeks, another episode of respiratory infection happened (day 18) (at the same time, half of the kindergarten group had respiratory symptoms, and a teacher had confirmed severe COVID-19 pneumonia). The patient had positive IgM against SARS-CoV-2, but the nasal swab PCR for SARS-CoV-2 was negative. 4 days of intravenous dexamethasone, accompanied with antibiotics, was given (Figures 1D,E). During the following 6 months (days 42, 64, and 137), she had three more episodes of respiratory distress (CT described the ground-glass opacities as affecting 70% of the lungs), requiring assisted lung ventilation through a tracheostomy tube, with febrile fever of up to 39.2°C and with good response to corticosteroids and antibiotics. After a while, the patient developed strabismus (days 64–80). The girl had constant hyperthermia within 37.6°C without laboratory signs of systemic inflammation.

The patient had features of metabolic syndrome: increased insulin level −207.4 pmol/L (n.v. = 17.8–173), with normal levels of blood glucose and HbA1c, dyslipidemia—triglycerides at 6.3 mmol/L (n.v. = 0.0–1.69), VLDL at 2.89 mmol/L (n.v. = 0.1–1.0), HDL at 0.63 mmol/L (n.v. = 1.04–1.55), and hyperuricemia at 585 mmol/L (n.v. = 150–350) associated with obesity. She had elevated IGF1 at 473–297 mcg/L (n.v. = 49–283). By the provided documents, the girl used to have secondary hypocorticism and hypothyroidism, which resolved over time (days 80–132). Her endocrine function was not evaluated before the COVID-19 infection since she was considered healthy. Proteinuria was intermittent since day 39, and electrolyte disorders persisted since day 46 [hypernatremia 155.8–162 mmol/L (n.v. 130–145 mmol/l), hyperchloremia 116.9–129.6 mmol/L (n.v. 98–107 mmol/l)]. At around the same time, central hypoventilation and bulimia started (days 45–63), explaining the recurrent chest infections.

The antinuclear antibodies, anti-MPO, and anti-PR3 antibodies were negative; immunoglobulins A, M, and G were normal, while the IL6 (10.49 pg/ml, n.v. <7) and C3 complement components (2.74 g/l, n.v. 0.83–1.93) were slightly elevated.

SARS-CoV-2 IgM remained positive over time, decreasing to borderline after 1 year; the erythrocyte sedimentation rate (ESR) and D-dimer remained elevated: ESR, 30 → 70 → 36 mm/h (n.v. = 2–15) and D-dimer, 1.22 → 0.72 → 3.05 mcg/ml (n.v. = 0.09–0.53).

At first admission to our hospital (days 229–268), the girl had hypercapnia (tcCO_2_, 40–60 mmHg); SpO_2_ was 97–100% while she was awake and 92–96% during sleep on assisted lung ventilation, with episodic desaturations to 72–86%. Unfortunately, polysomnography was not available until day 412, when central hypoventilation was proven. On admission, 7.5 months after the disease onset, she continued being obese (BMI, 33.3 kg/m^2^) and required assisted lung ventilation through a tracheostomy tube while asleep. She could sit without support, hold hands above her head for more than 10 s, roll prone to supine and supine to prone, and stand for a short time with support. The results of the Denver Development Screening test were as follows: MQ, 0.32 (n.v. >0.75) and DQ = 0.7 (n.v. ≤ 0.7). Her behavior issues include short periods of “aggression” directed at other people. Cranial innervation was intact despite periodically alternating divergent strabismus (as reported by the mother, the child had transient ptosis at the onset of the disease). The muscle tone was diffusely reduced, without asymmetry, the muscle strength [according to the Medical Research Council (MRC) Scale] was reduced to three points, and the tendon reflexes were alive. There were no meningeal and cerebral symptoms; the coordinating tests were optimal.

A repeat MRI of the brain and hypothalamic–pituitary region with contrast showed mild post-hypoxic changes. In contrast, COVID-associated acute disseminated encephalomyelitis, corpus callosum lesion, brain tumor, post-hemorrhage lesion, and ischemic stroke were ruled out as well as known immune-mediated CNS disorders and primary disease CNS–angiitis. The neurophysiological study did not suggest acute inflammatory demyelinating polyneuropathy and inflammatory or congenital myopathy. The electroencephalography result during wakefulness showed age-appropriate bioelectrical activity.

The chest CT showed interstitial changes and atelectasis in both lungs, which are attributed to hypoventilation.

The echocardiography and electrocardiography (ECG) results were normal, with normal ejection fraction and no signs of pulmonary hypertension, systolic or diastolic dysfunction, or other cardiac issues. During 24-h ECG and arterial pressure monitoring, reduction of heart rate variability and diastolic arterial hypotension were detected. Diastolic arterial hypotension, reduction of heart rate variability, constant hyperthermia within 37.6°C, strabismus, and central hypoventilation were considered autonomic dysregulation.

**Figure 2:**
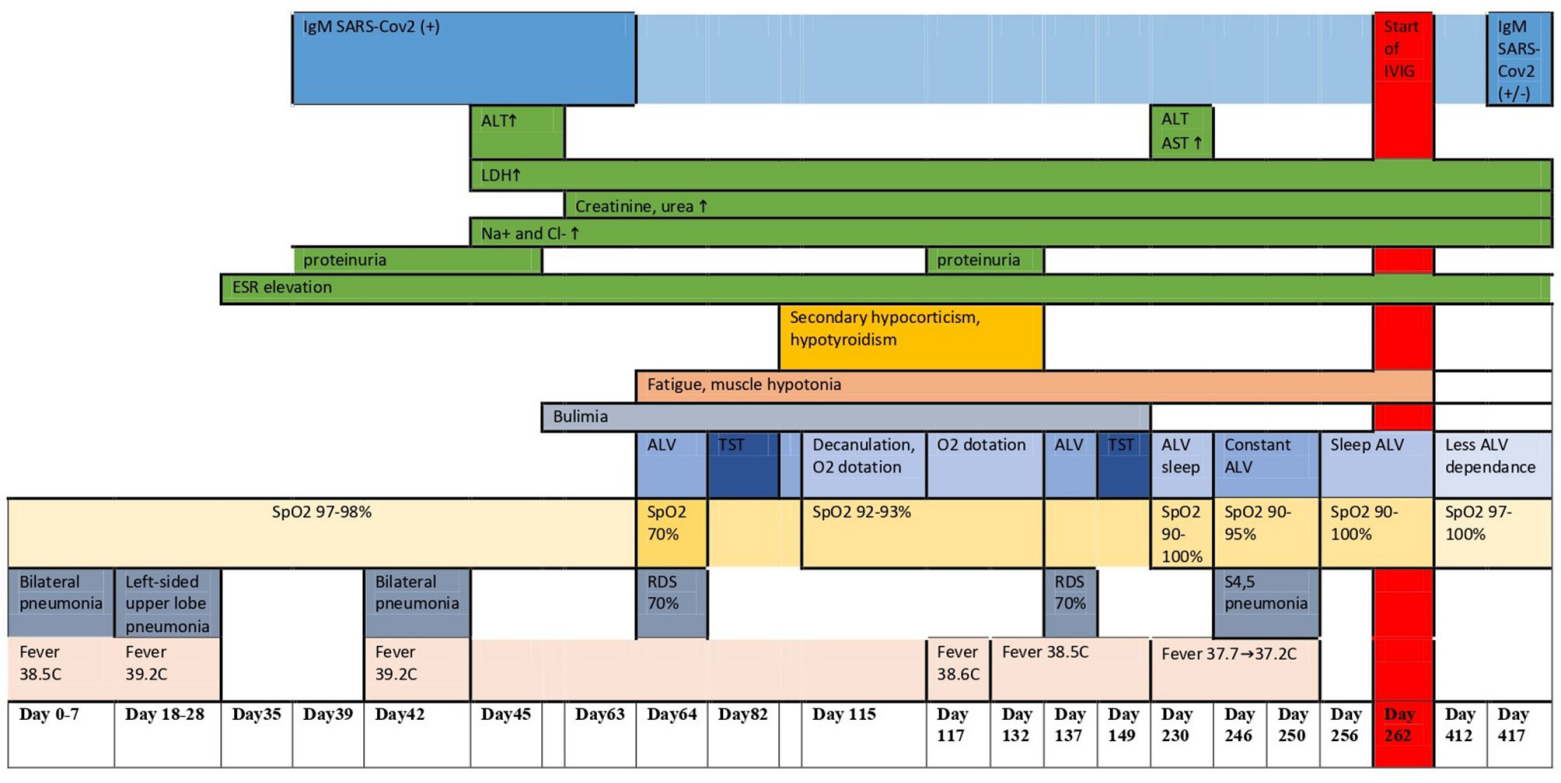
The whole disease course of the patient.

The karyotyping showed a normal female karyotype (46, XX). The Sanger sequencing and multiplex ligation-dependent probe amplification (MLPA) technology using SALSA MLPA Probemix P318 kit (MRC Holland, The Netherlands) did not find mutations in the *PHOX2B* gene. As a result, polyalanine repeat expansion mutations, non-polyalanine repeat mutations, and whole-gene or exon-specific deletions were excluded. The results of FISH with locus-specific DNA probe Prader-Willi/Angelman SNRPN (15q11)/PML (15q24) was also normal. Whole-exome sequencing is in progress. The clinical dynamics, laboratory findings, and imaging allowed us to define the condition as a ROHHAD-like syndrome. The disease course is depicted in Figure 2 and Table 1.

**Table 1:** The laboratory feature dynamics during the disease course.

	**Before admission D0-D229**	**First admission D229-D250**	**Deterioration D246-D250**	**Between admissions D313**	**Second admission D402-D418**
Uric acid, umol/L (n.v. 150–350)		393 (↑)			585 (↑)
GGTP, IU/l (n.v. 9–36)		56 (↑)			19
CK, IU/l (n.v. 29–168)		34	37		62
LDH, IU/l (n.v. 125–220)	453 (↑)	360 (↑)	525 (↑)		321 (↑)
AST, IU/l (n.v. 10–31)	33	41	26		27
ALT, IU/l (n.v. 10–31)	57	64–92	42		32.8
Creatinine, umol/l (n.v. 30.93–52.21)	50 (↑)	42.6	47.8		56.0
Urea, mmol/l (n.v. 2.5–6.0)	9.5 (↑)	8.6 (↑)	6.8 (↑)		7.2 (↑)
Glucose, mmol/l (n.v. 3.89–5.83)		4.5	4.6		4.6
Insuline, pmol/l (n.v. 17.8–173)	105.5			200.7 (↑)	207.4 (↑)
Potassium, mmol/l (n.v. 3.5–5.5)	4.6	3.8	4.0	4.1	3.8
Sodium, mmol/l (n.v. 130–145)	161	155.8	154.3	165	156.8
Chloride, mmol/l (n.v. 98–107)	125	122.1	125.3	132	120.2
TSH, mIU/l (n.v. 0.35–4.94)	0.498 (↓)	2.269		4.61	2.627
fT4, pmol/l (n.v. 9–19)	11.1			10.67	11
ACTG, pg/ml (n.v. 7.2–63.3)		21.71		45	35.27
Cortisole, nmol/l (n.v. 101.2–535.7)	234	314		404	172.5
LH, mIU/ml	<0.09	0.1		<0.09	0.1
FSH, mIU/ml	0.17	0.3			0.8
Prolactine, ng/ml (n.v. 4.79–23.3)	20.8	12.18		16.13	11.16
IGF1, ug/l (n.v. 49–283)		473 (↑)			297 (↑)
ESR, mm/h (n.v. 2–15)	24–8	30	77	27	36
C3 complement, g/l (n.v. 0.83–1.93)		2.74 (↑)			2.43 (↑)
CRP, mg/l (n.v. 0.0–10.0)	2.1	4.69	137.83	10	4.65
APTT, s (n.v. 28.6–38.2)		39.7 (↑)	42.1 (↑)		36.7
D-dimer, ug/ml FEU (n.v. 0.09–0.53)			1.22 (↑)		3.05 (↑)

*ACTG, Adrenocorticotropic hormone; ALT, alanine aminotransferase; APTT, activated partial thromboplastin time; AST- aspartate aminotransferase; CK, creatine kinase; CRP, C-reactive protein; ESR, erythrocyte sedimentation rate; FSH, follicle-stimulating hormone; fT4, free thyroxine; D, days; GGTP, gamma-glutamyl transferase; IGF1, insulin-like growth factor 1; LDH, lactate dehydrogenase; LH- luteinizing hormone; n.v., normal value TSH, thyroid-stimulating hormone; ↑, upper than normal range; ↓, lower than normal range*.

### Family History

**Figure 3:**
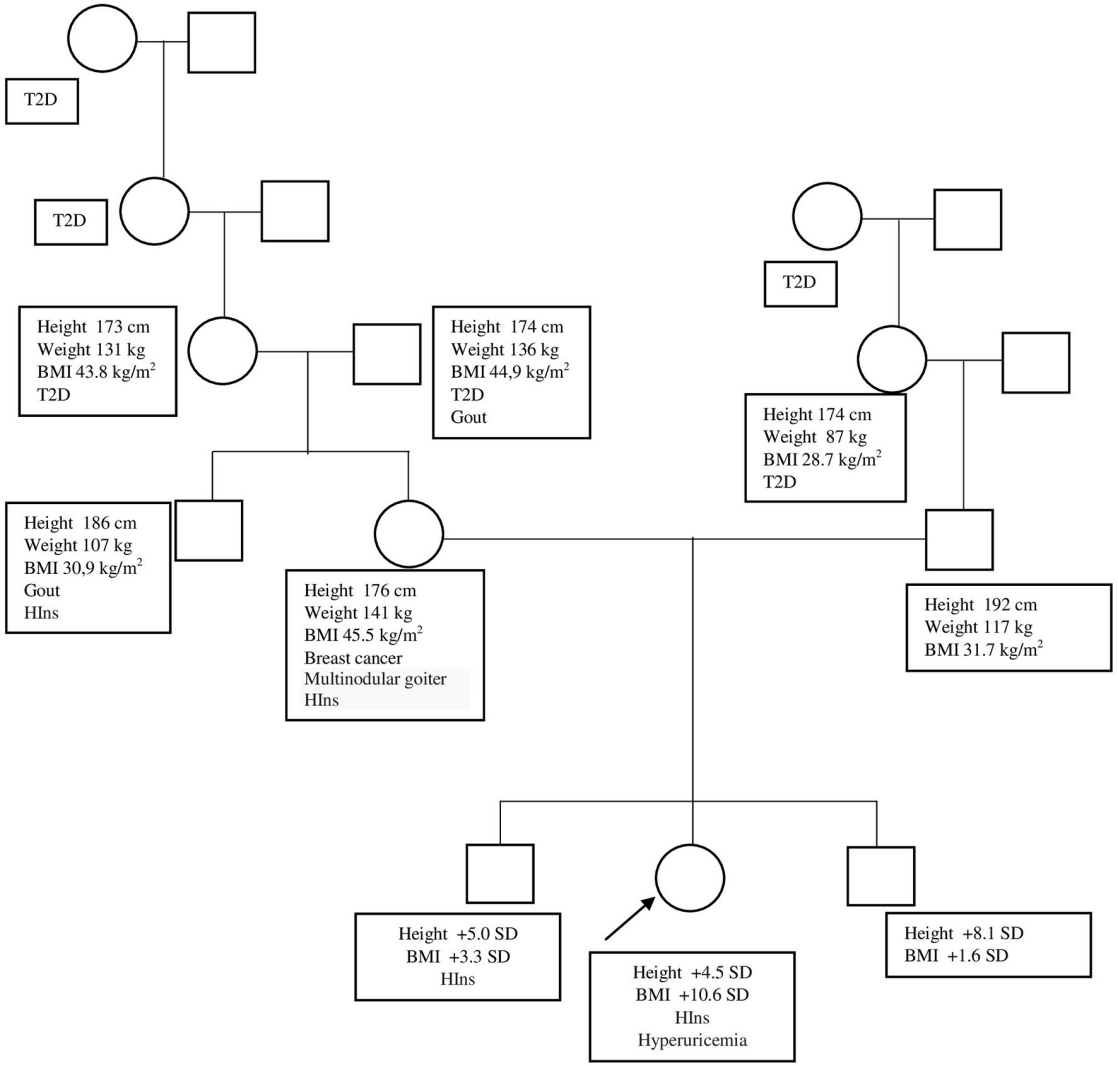
Patient's genealogic tree. BMI, body mass index; HIns, hyperinsulinism; SD, standard deviation; T2D, type 2 diabetes.

The Caucasian girl of Russian nationality is from non-consanguineous marriage. Both parents are tall and obese (mother's height, 176 cm; weight, 141 kg; BMI, 45.5 kg/m^2^; father's height, 192 cm; weight, 117 kg; BMI, 31.7 kg/m^2^). The girl's grandparents, the girl's uncle from her mother's side, and an elder brother are obese, while her younger brother, who is 1.5 years old, is tall for his age but not obese yet. Most of the relatives developed diabetes mellitus type 2 after 40–45 years of age (Figure 3).

### Therapy, Progress, and Outcomes

The anti-inflammatory treatment was started from day 262 [ibuprofen at 200 mg three times a day (11 mg/kg/day) for 2 months and monthly intravenous immunoglobulin (IVIG) at 1 g/kg of due weight, divided into two 2-h infusions once a day during 2 days] with dramatic clinical improvement.

**Figure 4:**
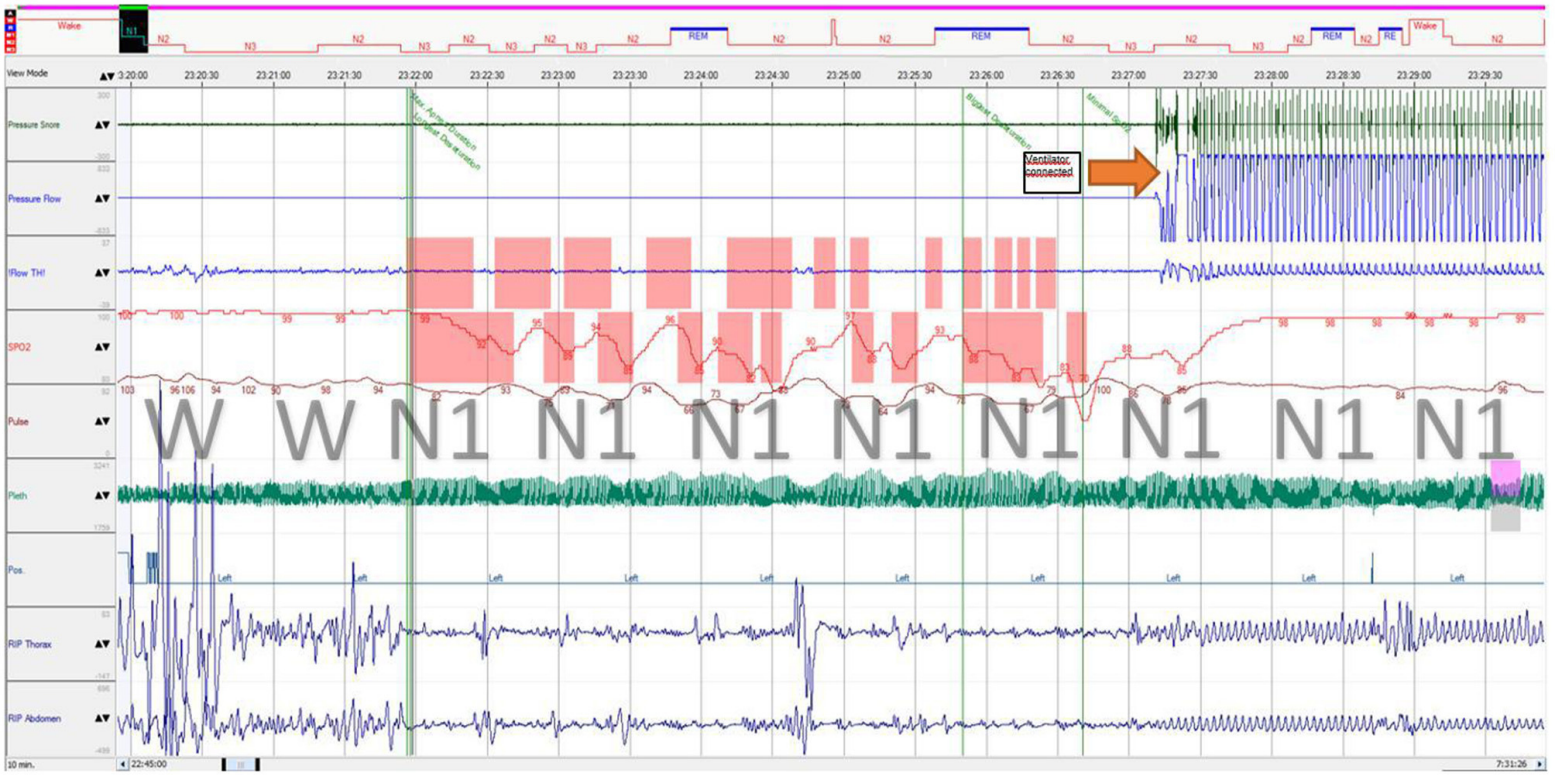
Polysomnography fragment of sleep onset with central breathing impairment followed by assisted ventilation. Immediately after sleep onset, the patient developed breathing movement amplitude decrease (both thoracic and abdominal) with a “periodic breathing”-like pattern associated with progressive desaturation and hypercapnia, which is considered central (airflow was not obtained from the tracheostomy tube during self-breathing). REM, stage rapid eye movement; N1, stage 1 non-rapid eye movement; N2, stage 2 non-rapid eye movement; N3, stage 3 non-rapid eye movement; RIP, respiratory inductance plethysmography; Flow TH, oral thermistor flow sensor; Pleth, photoplethysmography.

After 5 months of therapy, the Denver Development Screening test results were MQ = 0.62 and DQ = 0.72. She had minor muscle hypotonia, and her tendon remained normal. Muscle strength (MRC Scale) was five points in the proximal upper limbs and lower limbs and reduced to four points in the distal upper limbs. She can now walk and run without support, do yoga and pony riding, and climb a small climbing slide. Though the girl remains obese, her BMI decreased (day 229 + 10.59 SD; day 412 + 8.4 SD). The requirement for ventilation decreased (assisted lung ventilation through a tracheostomy tube only during night sleep), and central hypoventilation persists during non-rapid eye movement sleep (Figure 4). The inflammation laboratory signs improved as well. Her BMI slightly decreased at 33.0 (+8.4 SD). However, her ESR remained elevated (36 mm/h).

We planned to continue IVIG for another 3 months, following teleconsultation or in-hospital admission for clinical, instrumental, and laboratory evaluation and further treatment decisions (anti-inflammatory therapy discontinuation, prolongation, or escalation and assessment of ventilation needs).

## Discussion

The girl with a family history of obesity and redundant height, who had the same trajectory of excessive weight and height gain from birth, presented with additional ROHHAD-like symptoms temporarily related to COVID-19 infection. Though other viruses were not searched for, we consider the initial episode as a COVID-19 probable case based on epidemiological, clinical, and serological data. The patient had typical features of the ROHHAD syndrome: central hypoventilation, electrolyte and endocrine disorders, and signs of autonomic dysregulation (febrile temperature without systemic inflammation, arterial hypotension, and strabismus). Considering the girl's physical development prior to the disease family history, we speculate that obesity and tallness might have a genetic nature and do not seem to be wholly associated with respiratory and hypothalamic dysfunctions. On the other hand, a gap between obesity and other symptoms has been reported in some ROHHAD cases (5), and the SARS-CoV-2 virus might have been triggered following ROHHAD symptom presentation. Thus, the diagnosis of classical ROHHAD syndrome seems doubtful. The fluctuating course of the disease with improvement on non-steroidal anti-inflammatory drugs and corticosteroid therapy maintenance of humoral activity (increased ESR and C3) allows assuming the permanent course of the inflammatory process with immune-mediated damage of the hypothalamic–pituitary region.

The amount of data on nervous system involvement in COVID-19 survivors is increasing. Neurologic symptoms can be divided into five major groups: (i) encephalopathies with delirium/psychosis and cognitive impairment; (ii) inflammatory CNS syndromes including encephalitis, acute disseminated encephalomyelitis, and isolated myelitis; (iii) ischemic stroke due to a pro-thrombotic state; (iv) peripheral neurological disorders, including Guillain–Barré syndrome; and (v) other uncategorized syndromes, including autonomic dysfunction with fever, dyspnea, fatigue, and syncope, like in our case (1–3, 25–28). Some of the neurologic symptoms manifested immediately after or even before the lung disease, while in other cases, neurologic symptoms were developed about a month after the disease onset or even later (29). Interestingly, only some of the patients with symptoms of encephalitis had MRI changes and markers of inflammation in the cerebrospinal fluid, which made the diagnosis of COVID-19-associated CNS involvement difficult (1, 29–33). As with other neurotropic viruses, the fundamental question for SARS-CoV-2 infection concerns the relative contribution of viral infection *vs*. host response to the subsequent damage (26).

We could not find data of any single case of detection of SARS-CoV-2 PCR from cerebrospinal fluid (CSF) samples in pediatric patients. Concerns on the presence of SARS-CoV-2 in CSF include the absence of validated tests and appropriate timing of lumbar puncture (33).

In the analysis of CSF of adult patients with SARS-CoV-2 infection and neurological manifestations, SARS-CoV-2 RNA in CSF was detected in 2 of 58 cases (34).

The delayed time of presentation and the presence of autoantibodies let us speculate that, at least, in some cases the virus does not affect CNS itself but promotes a secondary autoinflammatory process, similar to Guillain–Barré syndrome, after COVID-19 infection (30, 35, 36).Since April 2020, a new multisystem inflammatory syndrome in children (MIS-C) was related to SARS-CoV-2 infection (37). More than 186 patients with MIS-C were described so far (38). The majority had cardiovascular system involvement and respiratory failure. Most of them had inflammatory laboratory picture (elevated ESR, C-reactive protein, D-dimer, and ferritin levels, anemia, thrombocytopenia, neutrophilia) (38). Neurological issues were described as well (33, 37). In their systematic review of neurological complications in pediatric patients with SARS-CoV-2 infection, Siracusa et al. (37) showed that most of the cases of CNS involvement in COVID-19 patients, including headache, altered mental status, seizure, muscular weakness, and meningism, happened in the course of the MIS-C. The minority of neurological issues were secondary to cerebrovascular involvement, and only sporadic cases had other reasons. Presuming the inflammatory pathogenesis of the condition, immunosuppressive treatment, including glucocorticosteroids, intravenous immunoglobulin, and anakinra, was tried with good effect (33), just as it happened in our clinical case. Nevertheless, unlike our case, only a short course of immunosuppressive treatment was enough (37).

According to MRI, the temporal lobes, cerebellum, thalami, hippocampus, and pons can be involved (1, 29, 31, 32). However, no reports of hypothalamus or hypophysis involvement have been previously published.

The SARS-CoV-2 virus, causing COVID-19, enters in pneumocytes through binding with angiotensin-converting enzyme 2 (ACE2) receptors. The ACE2 receptors are located in different tissues, including the lungs, pancreas, thyroid, testis, ovary, adrenal glands, pituitary, and hypothalamus. Theoretically, all the abovementioned tissues might be targeted by the SARS-CoV-2 virus with inflammation development and signs of organ involvement (32). SARS-CoV-2 has a lot in common with SARS-CoV, which is tropic to the hippocampus and hypothalamic region. Viral particles were found in endothelial cells and neural tissue in the autopsies of SARS-CoV patients (39). However, in SARS-CoV survivors, only central hypercorticism and central hypothyroidism have already been described (29, 32). Hypothalamic involvement might be presented with central diabetes insipidus, hypopituitarism, hyperprolactinemia, follicle-stimulating, luteinizing, adrenocorticotropic, thyroid-stimulating, and growth hormone deficiencies, and disorders of temperature regulation, sleep rhythm, emotions, and behavior (31). The clinical course of hypothalamitis perfectly corresponds to the classical ROHHAD syndrome. The direct viral involvement of the hypothalamic–pituitary region by SARS-CoV-2 could also be supposed, though it cannot be confirmed in the described case.

Though the etiology and pathogenesis of ROHHAD syndrome remain unknown, an autoimmune theory is widely discussed (6–9, 11–13, 16–24). There are three findings to support this theory: (i) presence of oligoclonal bands (8, 17, 24), B-lymphocytes (13), and specific anti-hypothalamus and anti-pituitary autoantibodies in the CSF (9); (ii) lymphocytic infiltration in the hypothalamus and midbrain (19, 23); and (iii) the association with other autoimmune disorders like celiac disease (13) and autoimmunity-predisposing HLA alleles (DQB1^*^0201, DQB1^*^0202, or DQB1^*^0302) (18).

However, all these findings were discovered in some, but not all, ROHHAD patients, which interfere with the heterogeneity of this syndrome. On the other hand, with COVID-19 encephalitis, MRI and CSF changes were not always present either (1, 29–32). In some COVID-19 encephalitis cases as well, some of the ROHHAD patients were treated with corticosteroids and IVIG with temporary improvement. However, the best results were obtained with high-dose cyclophosphamide treatment (4, 9, 13, 16, 17, 21, 24, 40, 41).

In our case, the timing of the autonomic dysregulation and endocrine disorders, which is approximately 4 weeks after the possible COVID-19 infection, may indicate an immune-mediated mechanism of the disease. The effectiveness of the anti-inflammatory therapy also support this theory.

The limitations of this case report can be considered as the absence of the initial polysomnography data, which does not allow us to indicate the timing of central hypoventilation onset, and the absence of the CSF analysis data (lumbar puncture was not done at the local hospital and was considered to be irrational at 7 months after symptom onset).

## Conclusion

This case report not only expands available data on the clinical manifestations of COVID-19 in a pediatric population but can also help to understand ROHHAD syndrome's nature and impact on its treatment strategies. Some patients with ROHHAD syndrome might have a viral or immune-mediated nature, so immune-modulating therapy (especially IVIG) might be a promising option. The case may not be conclusively attributed to COVID-19 infection and, at the same time, is rather a ROHHAD-like than ROHHAD syndrome itself. The case presented seems to be a subject for further understanding and follow-up.

## Data Availability Statement

The original contributions presented in the study are included in the article/supplementary material, further inquiries can be directed to the corresponding author/s.

## Ethics Statement

Ethical review and approval was not required for the study on human participants in accordance with the local legislation and institutional requirements. Written informed consent to participate in this study was provided by the participants' legal guardian/next of kin.

## Author Contributions

All authors contributed to manuscript revision, read, and approved the submitted version.

## Funding

This work was financially supported by the Ministry of Science and Higher Education of the Russian Federation (agreement number 075-15-2020-901).

## Conflict of Interest

The authors declare that the research was conducted in the absence of any commercial or financial relationships that could be construed as a potential conflict of interest. The handling editor OK declared past co-authorships with one of the authors MK and the absence of any ongoing collaboration with any of the authors.

## Publisher's Note

All claims expressed in this article are solely those of the authors and do not necessarily represent those of their affiliated organizations, or those of the publisher, the editors and the reviewers. Any product that may be evaluated in this article, or claim that may be made by its manufacturer, is not guaranteed or endorsed by the publisher.
